# Carbon sequestration via wood burial

**DOI:** 10.1186/1750-0680-3-1

**Published:** 2008-01-03

**Authors:** Ning Zeng

**Affiliations:** 1 Department of Atmospheric and Oceanic Science and Earth System Science Interdisciplinary Center, University of Maryland, College Park, USA

## Abstract

To mitigate global climate change, a portfolio of strategies will be needed to keep the atmospheric CO_2 _concentration below a dangerous level. Here a carbon sequestration strategy is proposed in which certain dead or live trees are harvested via collection or selective cutting, then buried in trenches or stowed away in above-ground shelters. The largely anaerobic condition under a sufficiently thick layer of soil will prevent the decomposition of the buried wood. Because a large flux of CO_2 _is constantly being assimilated into the world's forests via photosynthesis, cutting off its return pathway to the atmosphere forms an effective carbon sink.

It is estimated that a sustainable long-term carbon sequestration potential for wood burial is 10 ± 5 GtC y^-1^, and currently about 65 GtC is on the world's forest floors in the form of coarse woody debris suitable for burial. The potential is largest in tropical forests (4.2 GtC y^-1^), followed by temperate (3.7 GtC y^-1^) and boreal forests (2.1 GtC y^-1^). Burying wood has other benefits including minimizing CO_2 _source from deforestation, extending the lifetime of reforestation carbon sink, and reducing fire danger. There are possible environmental impacts such as nutrient lock-up which nevertheless appears manageable, but other concerns and factors will likely set a limit so that only part of the full potential can be realized.

Based on data from North American logging industry, the cost for wood burial is estimated to be $14/tCO_2_($50/tC), lower than the typical cost for power plant CO_2 _capture with geological storage. The cost for carbon sequestration with wood burial is low because CO_2 _is removed from the atmosphere by the natural process of photosynthesis at little cost. The technique is low tech, distributed, easy to monitor, safe, and reversible, thus an attractive option for large-scale implementation in a world-wide carbon market.

## Background

Atmospheric CO_2 _concentration has increased from 280 to 380 ppmv (parts per million by volume; a 35% change) since pre-industrial time, largely due to carbon emissions from anthropogenic fossil fuel burning and deforestation [1]. The emission rate of carbon from fossil fuel (oil, coal and gas) consumption is currently about 8 GtC y^-1 ^(10^15 ^g of carbon per year) [2] while the deforestation rate for the 1990s is estimated to be 1.6 (0.5–2.7) GtC y^-1^. The cumulative fossil fuel emission since 1800 is 330 GtC, but only about half of that remains in the atmosphere; the remainder absorbed by carbon sinks in the ocean and on land [1].

Fossil fuel emissions are projected to reach 9–20 GtC y^-1 ^by 2050 in the absence of climate change policies, according to a range of emissions scenarios [3]. Depending on how the current carbon sinks change in the future, the atmospheric CO_2 _concentration for the Special Report on Emissions Scenarios (SRES) A2 emissions scenario is between 450–600 ppmv by 2050, and 700–1000 ppmv by 2100, and global mean surface temperature may increase between 1.5–5.5°C [4], with related changes in sea-level, extreme events, and ecosystem shifts. Scientists have argued that severe consequences will occur once atmospheric CO_2 _concentrations reach between 450 and 600 ppmv [5-7]. Beyond this point, global climate change would be very difficult and costly to deal with [8].

Keeping the atmospheric CO_2 _concentration below 450–600 ppmv poses an unprecedented challenge to humanity. There are two main approaches: (1) to reduce emissions; (2) to capture CO_2 _and store it, i.e., sequestration. Since our economy depends heavily on fossil fuel, which comprises more than 80% of primary energy use, to reduce carbon emissions requires drastic changes in energy use efficiency and the use of alternative energy sources that are generally not economically competitive at present [9,10]. Even if advanced technologies such as hydrogen power and nuclear fusion become economical, the infrastructure switch will take many decades. It is thus very likely that at least some carbon sequestration will be needed in the near future to keep CO_2 _below a dangerous level.

Carbon sequestration involves two steps: (1) CO_2 _capture, either from the atmosphere or at industrial sources; (2) storage. Capture out of the atmosphere is assumed to be much more expensive because of the low CO_2_ concentration in the atmosphere relative to N_2 _and O_2_. For this reason, most current proposals seek to combine capturing CO_2 _with power generation, with several pilot power plants planned or underway [11]. The proposals for storing captured CO_2 _include pumping it into deep ocean where CO_2 _may react with water under the high pressure to form methane hydrates [12] or stays in CO_2 _lakes, burying carbon inside deep ocean sediments where conditions are even more stable than ocean bottom [13]. The technique that has been most seriously considered, is to store captured CO_2 _in geological formations such as old mines and deep saline aquifers [14]. There is also a spectrum of biospheric carbon sequestration methods, such as enhancing oceanic plankton productivity by iron fertilization, reforestation or altering forestry and agricultural management practices to maximize carbon stored in soil and vegetation, but the potential and permanence of these biospheric techniques have been unclear.

Here I suggest a biospheric carbon sequestration approach in which wood from old or dead trees in the world's forests is harvested and buried in trenches under a layer of soil, where the anaerobic condition slows the decomposition of the buried wood. This can be supplemented by selective cutting of other suitable trees. On the storage side, high-quality wood can also be stored in shelters for future use. In this technique, CO_2 _capture is done by the natural process of photosynthesis, and storage is low tech and distributed, thus attractive in two important aspects: cost and safety.

## Results

### Carbon sequestration via wood burial: a basic assessment

**Figure 1:**
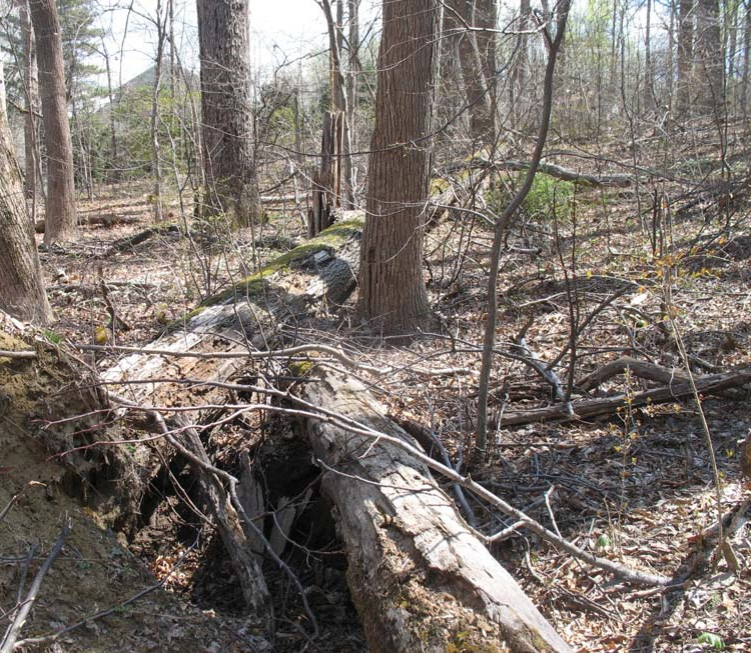
Dead trees on forest floor in a natural North American deciduous forest, Belwood, Maryland.

The possibility of carbon sequestration via wood burial stems from the observation that natural forest is typically littered with dead trees (Fig. 1). It is hypothesized that large quantities of organic carbon were buried and preserved for over one hundred thousand years under the great Northern Hemisphere icesheets during the Pleistocene glacial-interglacial cycles [15,16]. Other studies have shown that organic matter, especially wood, in municipal landfills decomposes extremely slowly [17]. With these, it became clear that wood harvesting and burial could be a viable method for carbon sequestration.

**Figure 2:**
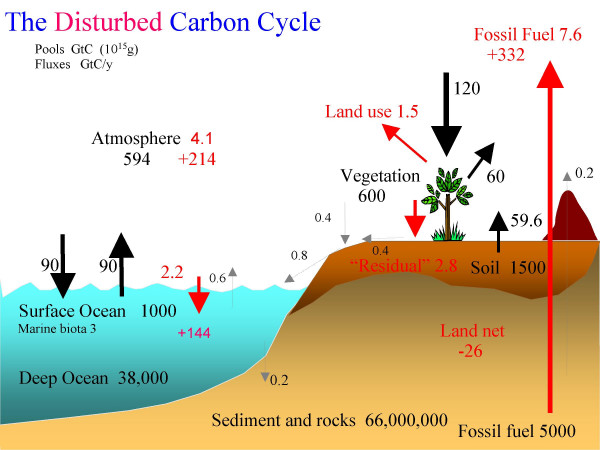
Major pools and fluxes of the global carbon cycle, with red color indicating anthropogenic fluxes for 2000–2006 and cumulative pools for 1800–2006 based on [40,41], with updates from [2]. About 1/3 (20 GtC y^-1^) of the net terrestrial productivity is wood production, a substantial fraction of which is the target of a sustainable carbon sink via wood burial.

Globally, approximately 60 GtC y^-1 ^are temporarily sequestered by land vegetation (Net Primary Productivity or NPP; Fig. 2). This carbon is continuously returned to the atmosphere when vegetation dies and decomposes (heterotrophic respiration, R_h_). In a steady state, the death rates of these carbon components equal to their respective decomposition rates and add up to NPP such that the net land-atmosphere carbon flux is near zero (NPP = R_h_). If we can stop or slow down a part of the decomposition pathway, we have the hope to sequester CO_2 _at a rate that may rival the current fossil CO_2 _emission of 8 GtC y^-1^. Since woody material is most resistant to decomposition due to its lignin-cellulose fiber structure which also minimizes nutrient lock-up (below), I will focus on this carbon pool.

Two major questions need to be first answered concerning the potential of this method: what is the production rate of dead wood, and how much is there in the world's forests? Unfortunately, there is a general lack of knowledge of dead wood on the forest floor, and this carbon pool is often neglected in carbon budget accounting. Since death rate is fundamentally limited by growth rate, the dead wood production rate can not exceed the world total NPP of 60 GtC y^-1^. Then the key question is how NPP is partitioned into the three main carbon pools: leaf, wood, and root. Leaves grow and fall in a deciduous forest each year, but may last a few years in an evergreen forest. Fine woody material such as twigs and small branches may break and fall often, but tree trunks and major branches have a lifespan of decades to centuries and longer. Thus, even though wood biomass is much larger than leaf biomass, its long lifetime suggests a production rate that is much smaller than otherwise. Root biomass can be large and the death rate is also substantial as roots constantly grow to search for nutrient and water. A 'naïve' first guess could be that NPP is partitioned equally into these three pools, leading to a 20 GtC y^-1 ^wood growth rate, thus 20 GtC y^-1 ^wood death rate at steady state. Since fine woody debris decompose more quickly and more difficult to handle, coarser material such as trunks and major branches are more suitable for burial. Assuming half of the woody material is coarse, then about 10 GtC y^-1 ^dead wood may be available for burial, thus leading to a 10 GtC y^-1 ^carbon sink. Assuming an average residence time of 10 years for dead trees on the forest floor, about 100 GtC (10 GtC y^-1 ^times 10 years) in the form of coarse woody debris would be already on the forest floor. These dead wood materials are under various stages of decay, but even if half of that can be collected and buried, it provides a substantial readily available carbon sink.

**Figure 3:**
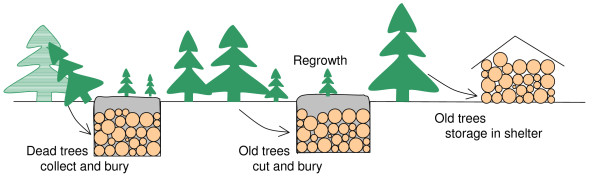
Schematic diagram of forest wood burial and storage.

The proposal is to (1) collect dead trees on the forest floor and (2) selectively log live trees. Then the tree trunks are either buried in the trenches dug on the forest floor (burial) or suitable landfills, or logs piled up above ground sheltered away from rain (Fig. 3). The buried woody material will have significantly longer residence time, and it effectively transfers carbon from a relatively fast decomposing pool (about 10 years) to a much slower carbon pool (100–1000 years or longer). In the case of (1), it reduces part of the heterotrophic respiration, and is thus an immediate effective carbon sink. In the case of (2), the subsequent regrowth in the 'gaps' left by tree cut is a carbon sink, which would depend on the rate of regrowth. In practice, (1) and (2) probably do not differ a lot, as fallen trees leave gaps for smaller trees to grow in a way very similar to case (2).

### Quantifying the carbon sequestration potential

#### 1 Sustainable sink of tree removal (limited by growth rate)

To quantify the size of this potential carbon sink, the global dynamic vegetation and terrestrial carbon model VEGAS [15,18,19] was used. While the model simulates the full terrestrial carbon cycle, only the carbon pools and fluxes relevant to the purpose here are discussed. The simulation did not include agricultural land, thus the estimates will be potential rates. The model was driven by modern observed climatology with seasonal cycles of precipitation, temperature, sunshine, wind speed, and vapor pressure. The simulation was run until convergence at a steady state where tree growth is balanced by mortality.

The modeled global NPP is 57 GtC y^-1^, of which 19 GtC y^-1 ^goes into dead leaf, 17 GtC y^-1 ^into dead wood, and 21 GtC y^-1 ^to dead root structures. Since fine wood (twigs and small branches) decomposes quickly, is more difficult to handle (more costly to clean up the leaves, etc.), and may occupy more burial space, only coarse wood will be considered as suitable for burial. Forestry literature generally makes a distinction between fine and coarse woody debris, typically using 10 cm stem diameter to separate the two classes. Unfortunately, the relative contribution to the total wood death from fine and coarse wood is difficult to quantify, in part due to the different lifetime (smaller stems generally have shorter life than the whole tree). It is sometimes unclear how these pools and fluxes are defined and what the reported numbers represent in forestry literature. I thus somewhat arbitrarily designate the fine:coarse ratio of death rate to be 7:10 so that the coarse wood death rate is 10 GtC y^-1^.

**Figure 4:**
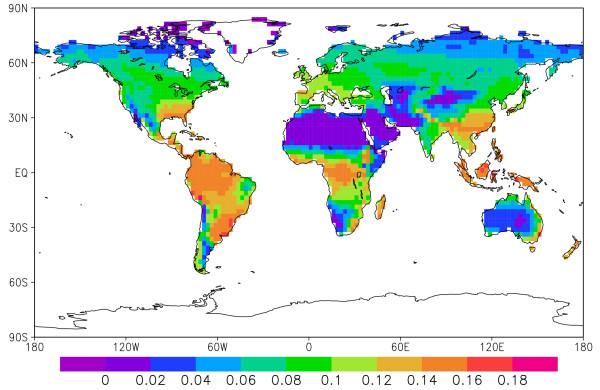
World coarse wood production rate estimated by the model VEGAS in kgC m^-2 ^y^-1^.

The spatial distribution of coarse wood death rate is shown in Fig. 4. The highest rate is found in the tropical rainforest such as the Amazon and the Congo basins, followed by temperate and boreal forests. The fact that the spatial distribution of wood death rate is similar to that of production (NPP) is not surprising because the death rate largely follows growth rate. Any regional deviation from the global mean partitioning ratio among the three pools (leaf:wood:root = 19:17:21) is the result of plant functional type (PFT) and climate dependent carbon allocation strategy. Such deviations are no more than 10–20% in this model.

The carbon sequestration potential of coarse wood for various geographical regions is given in Table 1. The tropical forest has a 4.2 GtC y^-1 ^carbon sequestration potential, temperate forest has 3.7 GtC y^-1^, while the boreal region has 2.1 GtC y^-1^. Since the model considers only potential vegetation (no agriculture) the temperate regions may have substantially smaller potential.

**Table 1:** Carbon sequestration potential based on coarse wood production rate (GtC y^-1^) estimated by VEGAS assuming potential vegetation for the main regions of the world.

Global	Tropics	Temperate	Boreal
10	4.2	3.7	2.1

At a regional scale (Table 2), South America has a carbon sequestration potential of 2.3 GtC y^-1^, with major contribution from the Amazon rainforest. Africa follows with 1.9 GtC y^-1^. Russia has a potential of 1.2 GtC y^-1 ^due to its vast expanse of boreal forest. The conterminous US has a potential of 0.8 GtC y^-1 ^with its extensive broadleaf and mixed forests along the East Coast and the South, and the mountainous West. Canada has a 0.7 GtC y^-1 ^potential from its mixed and boreal forests. Of the 0.9 GtC y^-1 ^potential for China, probably only a fraction can be realized because much of the country's forests has long been converted into cropland. However, a successful reforestation program could boost the size of this fraction.

**Table 2:** As in Table 1, but for some sub-regions (may overlap).

N Am	US	Canada	S Am	Africa	Europe	Russia	Asia	China	SEAsia	AusNZ
1.5	0.8	0.7	2.3	1.9	0.7	1.2	1.8	0.9	0.6	0.4

The coarse wood death rate estimated by the model is the result of plant functional type and climate dependent carbon allocation strategy that is not well constrained in current generation of global vegetation models [20]. Observations on this carbon pool and its turnover rate have been generally lacking. Nonetheless, some research has emphasized the importance of this carbon pool. Using observed and estimated average tree mortality rates and extrapolating point data using global biomass distribution, Harmon et al. [21] estimated the production rate of coarse woody debris to be 2–11 GtC y^-1^, with the uncertainty range coming from the tree lifespan estimates. Based on [21], Matthews [22] estimated 6 GtC y^-1 ^as the coarse woody debris production rate. A comparison is listed in Table 3. Thus VEGAS model result is within the range of [21] but on the high side. One of the reasons may be that the equilibrium simulation of VEGAS implies that the modeled forests have reached a steady state, i.e., they are mature forests, while the data used include forests of different ages. Since younger forests tend to have lower mortality than old-growth ones, these young forests will have higher potential in the future as mortality rate increases towards maturity. Given the many unknowns in both methods, I will assign a factor of 2 uncertainty to the 10 GtC y^-1 ^model estimate, i.e., a range of 5–15 GtC y^-1^.

**Table 3:** A comparison of estimates of world total coarse wood production rate (GtC y^-1^) and coarse woody debris (GtC).

	Harmon et al., 1993	Matthews 1997	VEGAS(this study)
Coarse wood production rate	5 (2–11)	6	10 (5–15)
Coarse woody debris	60–232	75	130

In estimating the 10 GtC y^-1 ^potential, I assumed natural vegetation, which by itself would be an overestimate because some of the potential forest area has been converted to cropland. Since current world forest area is 3 times that of cropland, and a significant part of cropland corresponds to potential grassland and even desert rather than potential forest, the degree of overestimation is modest. On the other hand, the actual potential could be higher due to other factors such as selective cutting (below), planting fast growing tree species, and burying smaller-sized wood. In addition, reforestation, deforestation and climate change in the future will complicate any attempt at a precise estimate including land use. Thus, the choice in using potential vegetation was made here.

#### 2 One time potential from existing coarse woody debris

**Figure 5:**
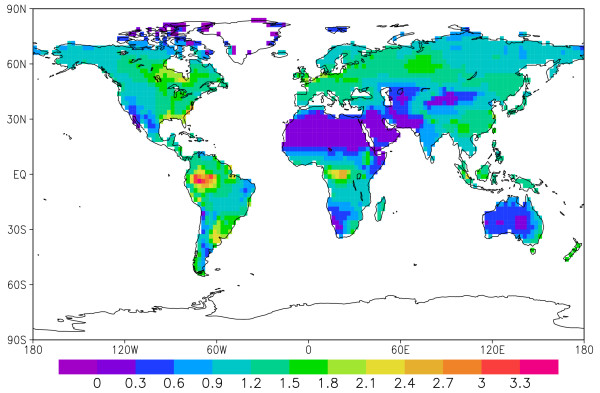
World distribution of coarse woody debris, in kgC m^-2^.

As a legacy of past tree death, a significant amount of dead wood has accumulated in the world's forests in various stages of decay (Fig. 5). I used the model VEGAS to simulate this dead wood pool and estimated global coarse woody debris to be 130 GtC, somewhat larger than the estimates of 75 GtC of [22], but within the range of 60–232 GtC estimated by [21]. These numbers may seem large as relatively little attention has been paid to this carbon pool, but they are not surprisingly large in light of the 390 GtC stored in world's forest vegetation biomass (mostly wood; [23]). Since wood at later stages of decay is not suitable for burial (also less likely to be included in forest inventory studies), even if half of this pool is suitable for burial, that is still 65 GtC available for sequestration. The spatial pattern (Fig. 5) shows a somewhat different distribution from the production rate with higher values in temperate and boreal region mostly due to the slower decomposition rate at lower temperature.

The implication of this large existing carbon pool is that in the initial stage of wood burial, more than the sustainable rate of 10 GtC y^-1 ^estimated above will be available.

#### 3 Enhancing the sustainable rate via selective cutting of live trees

The 10 GtC y^-1 ^dead wood production rate could also be enhanced by active forest management. Instead of waiting for the trees to die, one can also harvest relatively mature trees via techniques such as selective cutting. At first sight, this seems to be a carbon source as live trees take up CO_2_. However, if trees are selected properly, it may lead to an overall sink because younger forest tends to be more productive, and somewhere in the development stage, productivity significantly exceeds respiration and decomposition loss [24]. Since the less productive trees that do not do well compete for light and other resources, their removal will leave younger trees to grow more vigorously in the gaps, forming a net carbon sink. In an even-aged forest, self-thinning is a major step of the secondary succession in which a major fraction of young trees die to give way to other trees. In this case much younger trees can be selectively cut or collected after death.

### Implementation strategy

The implementation of a wood burial scheme will involve three major steps:

(1) Enabling access to the forest if not already in place;

(2) Site selection, trench digging for burial or building a shelter for above ground storage;

(3) Selective tree cutting or the collection of dead wood followed by trimming, shortening and burial or storage, repeated at an appropriate return interval.

**Figure 6:**
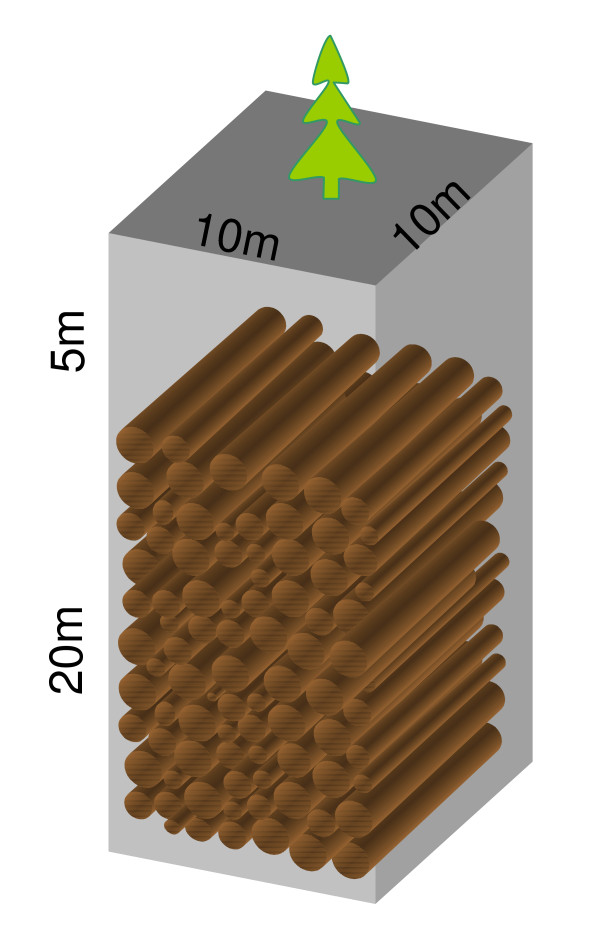
An example trench that could bury 500 tC, the amount of coarse wood carbon from a typical midlatitude forest area of 1 km × 1 km in 5 years.

I envision a network of roads and paths that will allow machine access, and trenches that are distributed at a more a less uniform spacing. For example, a 1 km × 1 km area (100 hectares) would accumulate about 100 tonne of carbon per year for a typical coarse wood production rate of 0.1 kgC m^-2 ^y^-1 ^(Fig. 4). At a return interval of 5 years, each trench would bury 500 tonnes of carbon (about 1000 tonne dry wood mass). Assuming a 0.5 tonne dry matter per cubic meter and neglecting some space in between the logs, the volume required would be 2000 m^3^. If the pile is buried under 5 meters of soil, the trench can have the dimensions of 10 m × 10 m × 25 m (Fig. 6). The surface area would be 100 m^2^, only 0.01% of the wood collection area, thus the disturbance would be small. Soil will fill the space in between logs and above and be allowed to settle. Vegetation can be allowed to grow back naturally on the burial sites. Selective sites can be monitored for the decay of the buried wood. Figures 3 and 6 illustrate these procedures.

The actual trench size and distribution need to balance several factors such as cost of digging trench, transporting deadwood, minimizing disturbance to the forest, and selecting the location that most effectively prevents decomposition. Onsite burial is preferred wherever possible to minimize transportation cost. Transportation may be needed where soil is too shallow to dig trenches of sufficient depth. Since soil condition can vary greatly even within a small area such as soil moisture content variation associated with topography, care needs to be taken in site selection.

Depending on the dead wood accumulation and decay rates, this process can be repeated every few (1–10) years, but the burial sites will be different each time. The main criterion for choosing return interval will be a balance between the cost of each operation and the need not to let the dead trees rot away. If selective cutting is the main operation mode so that there is little natural tree death (trees are cut before they die), the dominant factor will be the density of suitable trees to remove. In the case of plantation, it may be a good strategy to clear cut small sections (group cutting) for its low cost, allowing trees to grow back as secondary succession.

Compared to above-ground shelter storage, trench burial is a better choice for fallen trees as they are typically already in the process of decomposition, so they are less useful as lumber wood. On the other hand, shelter storage preserves lumber wood for easy use should future demand increases.

The technology required for collecting or selectively cutting trees is low tech and has been around for thousands of years. Most modern large-scale logging is done by machines in many places such as Europe and North America. The road system for access is already in place in many of these regions such as the US 'Forest Highway' system. Half of the world's forests are already within 10 km, and three quarters are within 40 km of major transportation infrastructure [25]. Since there is no major technological hurdle, such a scheme can be implemented almost immediately in a substantial fraction of these regions. For instance, a common practice in North American forestry is to hire private logging companies with a variety of operation scales to cut trees on private or public land, allowing the flexibility of handling forests of different sizes and conditions. Although currently intensely managed forests have little dead wood immediately available for burial, their long-term potential still holds.

Such a distributed system can be run with little government intervention except for monitoring, as long as economic incentive is provided through schemes such as carbon trading. In North America, much of the forested land is privately owned. The potential for carbon sequestration will have a positive impact on the logging industry and many land owners and the economy in many regions. The accounting and monitoring of the carbon sinks can be done by certified engineers when logging companies return for each round of harvest. This can be supplemented by larger-scale monitoring systems such as eddy correlation flux measurement [26], source/sink inversion using atmospheric CO_2 _measurements [27] assisted by future satellite CO_2 _observations [28]. The vast expanse of boreal forests in Canada and Eurasia are only partly accessible and largely unmanaged at present, but infrastructure such as roads can be built relatively quickly in the relevant countries.

If a major portion of the estimated 10 GtC y^-1 ^carbon sequestration potential is to be utilized, nearly all the world's forests will need to be managed. Then a main question would be the accessibility to the remote forest regions. Firstly, extremely steep mountainous regions or boggy wetland will be difficult to access. Secondly, there are practically no roads to the deep tropical forests. Moreover, a proposal of building a network of roads in the heart of a rainforest will raise major environmental concerns such as loss of biodiversity. On the other hand, economic incentives will continue to stimulate such road expansion. Even in this case, the issue of law enforcement for illegal deforestation, and more broad governance issues need to be first ensured before countries in these regions reach a point-of-no-return. In the near future, a beneficial practice is to bury rather than to burn the trees in the regions with ongoing deforestation.

If the cores of the tropical rainforests are to be left intact which accounts for about 20% of the total carbon sequestration potential (half of the tropical rainforest; Table 1), sequestration in the remaining tropical, temperate and boreal regions still provide a sink of 8 GtC y^-1^. Difficulty in accessing steep terrains where forests are typically better preserved will further reduce this number. In fact, giving the cost of road construction and environmental concerns, it is desirable to manage more efficiently a smaller fraction of the available forests through methods such as selective cutting or burying part of the finer woody debris, than disturbing a larger fraction at lower per unit area carbon sequestration rate.

### Cost

The scale of the climate change problem dictates that any mitigation strategy, whether being alternative energy source, carbon sequestration technique, or geo-engineering approach, has to be cost effective when operated on a large scale. Data from the US logging industry indicate that a typical cost for harvesting 1 tonne of lumber wood is about $20 [29]. Since lumber wood is only part of the coarse woody material that can be buried, which I assume is about 50% more than lumber wood alone (there are substantial amount of smaller branches compared to the trunk). In the other direction, given that lumber wood contains some water and that plant dry mass is approximately 50% carbon, the cost could be $40 per tonne of sequestered carbon. This would be higher if the cost of trench digging, road construction and maintenance is included. I will thus put the cost at $50 for 1 tC (tonne or 10^6 ^gram of carbon) sequestered, with an uncertainty range of $25–$100/tC.

It is illuminating to compare this with power plant CO_2 _capture and geological storage (CCS; Table 4), a strategy that has been under intense study [14]. The $50/tC ($14/tCO_2_) cost for wood burial is lower than the $20–270/tCO_2 _for power plant CCS. The large range in power plant CCS is due to the increasing cost as cheaply available old mines run out. In the case of wood burial, there is no practical storage capacity limitation. A major cost of industrial CCS is the capturing of CO_2 _from flu gas, while wood burial is free air capture with near-zero cost because it is done by the natural process of photosynthesis.

It is also interesting to compare this cost with the pioneering European Union Emission Trading System (EUETS) carbon cap-and-trade market price. The EUETS price has fluctuated between €1–33/tCO_2 _during 2005–2007. In comparison, the voluntary Chicago Climate Exchange (CCX) price has been around $3–4/tCO_2_. Although the wood burial cost is somewhat higher than the current market price, it is expected that future climate mitigation policy will result in higher prices for carbon. When implemented at global scale, many factors will vary from location to location such as technology and labor costs. The cheapest will be the forests that are already under intense management where roads and machinery are in place. The price may increase as the total area of forests utilized this way increases. The operation of machinery will consume some fossil fuel and emit CO_2_. These factors need to be evaluated.

**Table 4:** Comparison of wood burial and power plant CCS. The markets use tCO_2 _as carbon unit which can be converted into tC with the conversion factor the molecular weight ratio CO_2_:C = 44:12; both units are shown.

Wood Burial	Power plant CO_2 _capture with geological storage	Price on Chicago Climate Exchange (CCX) 2006	European carbon trading market price during 2005–2007
$14/tCO_2 _($7–27)	$20–270/tCO_2 _[14]	$3–4/tCO2	€1–33/tCO_2_
$50/tC ($25–100)	$73–990/tC	$12–16/tC	€4–120/tC
Storage safe; semi-permanent, reversible; some environmental concern	Possibility of leakage; lower cost storage capacity small		
Potential: 10 ± 5 GtC y^-1^ Long-term: thousands of GtC or no practical limit	Potential rate is limited by scale of operation Longterm: > 500 GtC		

### Scale of operation

Even if only half of the estimated potential (5 GtC y^-1^) is carried out in the next few decades, say, by 2050, the scale of such a world-wide operation would be enormous, as illustrated in the scenario below.

If each trench has a 500 tC capacity (example in Fig. 6), then the number of trenches needed for a 5 GtC y^-1 ^sequestration rate would be 10 million per year, i.e., one trench every 3 seconds. Assuming it takes a crew of 10 people (with machinery) one week to dig a trench, collect/cut and bury wood over a 100 hectare area, 200,000 crews (2 million workers) and sets of machinery would be needed. This estimate is admittedly simplistic and the task could be quite labor-intensive if it is to be carried out in dense or steep-sloped natural forests.

The scale of such an operation may be difficult to imagine at first sight, but the enormous scale of the CO_2 _problem means that any effective mitigation strategy also has to be at a comparable scale. The current rate of 8 GtC y^-1 ^fossil fuel carbon emission rate corresponds to 250 tC per second. Since carbon content of wood is roughly the same as in fossil fuel, if wood burial is to counteract the fossil fuel emission (as it could potentially do), the rate (in terms of either mass or volume) at which we bury wood needs to be comparable to the rate we burn fossil fuel. If wood burial is used as part of a portfolio, the operation could be scaled down accordingly.

The plausibility of this operation may be more easily comprehended from an economical point of view. A $50/tC cost for wood burial corresponds to $250 billion per year at a 5 GtC y^-1 ^sequestration rate. This is only 0.5% of world total Gross Domestic Product (GDP) of $48 trillion in 2006, compared to the projected 5–20% GDP potential economic damage from climate change [8]. The $250 billion per year cost for 2 million workers means $62,500 per worker, assuming half is for machinery and other costs. Obviously, labor and machine costs can be very different in different countries. The job opportunities provided by the operation and other positive impact on the economy will be attractive in many regions especially the developing countries.

## Discussion

### Potential issues

#### 1 Decomposition of buried wood

Because of the low oxygen condition below soil surface, the decomposition of buried wood is expected to be slow. This is supported by the observation of extremely slow decomposition of woody material such as furniture in landfills where wood products are found to be well preserved after many years of burial by Micales and Skog [17]. Indeed, these authors found that only 0–3% of the carbon from wood are ever emitted as landfill gas after several decades, and considered the remaining fraction locked away 'indefinitely'. Ancient wood can be preserved for thousands of years in undisturbed archeological sites. Indeed, the current proposal can be viewed as creating 'graveyards' for dead trees worldwide. In the boreal forests where the temperature is low, decomposition can be very slow as evidenced by tree trunks hundreds of years old on the boreal forest floor. Since decomposition rate is also function of moisture, the burial sites need to be chosen properly in consideration of local topography and hydrology. If needed, the decomposition could be further slowed by sealing the outer layer with resistant material such as wax. It is also possible to bury dead wood in wetlands or under water, but there will be major transportation cost, availability of suitable sites, and permanence issue in face of human activities and climate change.

**Figure 7:**
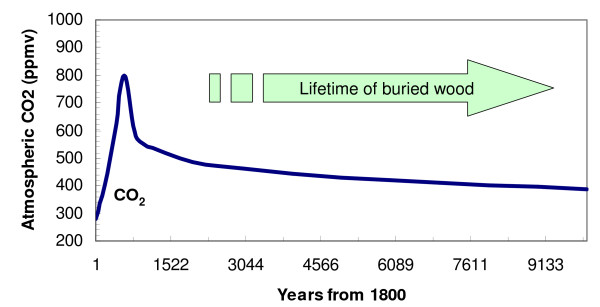
Lifetime of buried wood can be substantially longer than fossil fuel CO_2 _residence time in the atmosphere. CO_2 _concentration is based on a scenario in which 1000 GtC fossil fuel is burned in the next few hundred years.

The 0–3% range of decomposition rate [17] translates into an e-folding timescale of 1000 years to infinity, assuming a 30 year average age for landfills in their survey. If these burial sites are better protected through, e.g., thicker soil cover, the preservation would last even longer. Thus, we can slow down the decomposition rate of collected wood at least to the timescale of 1000 years (most likely longer) so that the release of this buried carbon pool is negligible compared to forest regrowth uptake in response to collection/cutting that occur on the timescales of decades. If the buried carbon comes out slowly over the timescale of thousands of years, it should have already passed the major peak of atmospheric CO_2 _as the anthropogenic CO_2 _'pulse' is absorbed into the deep ocean and the carbonate sediments (Fig. 7; [30]).

Depending on the burial depth, the deep roots of trees re-growing on some burial sites may eventually invade into the trench and facilitate the decomposition of buried wood so that the nutrient and carbon will slowly return to the surface and the atmosphere. Although the vegetation could be made not to re-grow above the trench, or the buried wood could be insulated from the top soil by a layer of resistant material, re-growth might be more desirable than 'permanent' burial (tens of thousands of years or longer). Thus the way wood is buried will determine the decomposition rate, and can be managed to desired effect. Long term monitoring and research of representative sites will be useful for finding optimal burying methods.

#### 2 Nutrient lockup

One potential drawback of wood burial is that nutrient in wood will be locked away. The same drawback also applies to other methods of large-scale vegetation use such as biofuels. This is a serious concern because nutrients may already be a limitation for plant growth in some forest ecosystems. Plants recycle a major part of the nutrient in dead material. This is especially so in the tropical rainforests where the recycling is so efficient that most of the nutrient is locked in live and dead trees rather than in the soil. If a major fraction of the nutrient becomes locked up by buried wood, the forest growth could be severely limited after some decades so that the strategy becomes unsustainable. Here I use nitrogen as an indicator of nutrient for analysis.

Fortunately for our purpose, the nutrient content in wood is much smaller than in leaves and fine roots. For instance, typical carbon to nitrogen ratio (C:N) is 20:1 for leaves, but 200:1 for wood [31,32]. This is fundamentally because the structural components of plants consist mostly of lignin-cellulose complexes which are carbohydrates, i.e., C, O and H, while nutrients are concentrated in the photosynthetic and metabolic components such as chlorophyll and protein.

The magnitude of this potential problem can be viewed in two ways. First, because leaf turnover rate is comparable to wood turnover (above), but the C:N ratio is 10 times larger, so that the nitrogen recycling rate in leaves and fine wood is more than 90% faster than that in the coarse wood, even though the total amount of nitrogen in wood may not be too different from that in leaves. The fact that tropical rainforest is extremely quick at 'grabbing' whatever nutrient is on the forest floor suggests the great ability of forest at utilizing what is on the ground.

The ultimate question is whether internal fixation and external input are fast enough to compensate for the loss rate due to burial lockup. If 10 GtC y^-1 ^of carbon is to be buried, a C:N ratio of 200 implies that about 50 MtN y^-1 ^(Mega tonnne or 10^12 ^gram of nitrogen per year) will be locked up in the buried wood. Although 50 MtN y^-1 ^is a nontrivial amount, this is only a fraction of both the global natural nitrogen fixation rate of 110 MtN y^-1 ^and the anthropogenic N (mainly from fossil fuel burning and fertilizer use) deposition rate of 140 MtN y^-1 ^[33]. In addition, natural fixation rate may increase when nitrogen is in short supply. Thus, globally speaking, the nutrient lock-up due to burial does not appear to be a problem big enough to hold back the wood burial proposal. However, it will depend on the spatial distribution and the fraction of the nitrogen deposition that can be utilized [34]. Our current understanding of such issues is limited, and more research in this area is needed. In some regions or localities this may be a more important issue. In these cases, some moderate fertilization could be used to alleviate the problem, or the intensity of the operation could be reduced.

#### 3 Habitat loss

Dead wood, whether standing (snags) or down, plays an important role in forest ecology, acting as habitat for animals such as cavity-nesting birds, plants and microbial lives. To minimize the impact, it may be desirable not to completely clean the forest floor, but leave a fraction to maintain these important ecological functions.

#### 4 Disturbance to forest floor and soil

Although modern forest logging practice has shown that disturbance can be kept at minimum, there is no guarantee it will be the case when practiced world-wide. If not executed properly, it may harm forest regeneration capability, biodiversity and cause significant loss of soil carbon. One method is to have ecological monitoring and carbon accounting conducted together by certified agencies or institutes following carefully crafted international standards.

The soil carbon pool is a dynamic balance between dead vegetation input and decomposition. If the deadwood input to soil is reduced, the soil carbon pool will decrease somewhat. It is difficult to quantify this possible loss at present. Regardless of the extent of this soil carbon loss, equilibrium will be reached after sometime so that the cumulative effect of a sustainable wood burial will eventually exceed the initial loss.

#### 5 Competition with other wood usage

Wood has been a major resource for humans ever since our ancestors learned to use fire and sticks. Current world total wood consumption is about 0.9 GtC y^-1 ^[35]. Obviously, priority will be given to these uses such as furniture and building material, but compared to the 10 GtC y^-1 ^coarse wood production rate, there will be large additional capacity for carbon sequestration. Indeed, the burial scheme may be carried out most naturally as an expansion of the existing logging capacity. In addition, if old furniture and building lumbers are buried rather than left to decay in open dumps, they will still serve the purpose of carbon sink. This has already been practiced to some extent in landfills.

Research is ongoing in cellulosic biofuel where cellulose in woody material is converted to fuel [36]. Should this become economical with minimum environmental impact in the future, obviously it will have priority over wood burial because of the energy produced. This can also be said for other uses such as co-firing of wood chips and agricultural residue with coal. Nevertheless, the capacity built for wood harvest and burial will lend itself naturally to collecting wood for biofuel use. The 10 GtC y^-1 ^wood production rate also provides an (approximate) upper limit on how much biofuel can be produced, and the caveats discussed here such as nutrient lock-up also apply.

#### 6 Other unintended consequences

One possibility is that if roads are built into remote forests, it will make it easier for deforestation. What has happened in the Brazilian Amazon over the last 3–4 decades where deforestation (legal and illegal) follows road construction cautions against the implementation of wood harvest and burial in such regions. For this and many other environmental concerns, a considerable fraction should be preserved and left completely natural. A wise strategy would be intense management of suitable land to achieve higher efficiency while preserving as many forests in their natural states as possible.

There may be the concern that wood burial (or any other effective carbon sequestration scheme) will hinder the motivation to reduce emissions and the development of alternative energy. While this is a legitimate and important concern, there is currently a major mismatch between the urgency of the climate problem and the slow pace of the transition toward a carbon-neutral economy due to technological, economical and political hurdles. Carbon sequestration should only be used to 'buy time' so that the society has sufficient lead time to adjust while avoiding dangerous climate change.

### Synergy with other activities

#### 1 Reforestation and afforestation: making the carbon sink long-lasting

Reforestation is a widely embraced carbon sequestration technique [37,38]. However, its capacity in sequestering carbon is limited by competition with other land use purposes such as agriculture. In addition, as forest and underlying soil mature, the carbon sink becomes saturated. If the trees are cut or burned by fire, the stored carbon would be lost back into the atmosphere. Such concerns had led to a disappointingly small role of reforestation in the Kyoto Protocol under the United Nations Framework Convention on Climate Change (UNFCCC). Wood harvest and burial comes most naturally to such forests because they are by definition managed. Reforestation followed by wood burial will extend the lifetime of such land carbon sink indefinitely. Because much marginal land suitable for reforestation is currently not utilized, the earlier such activities are undertaken, the earlier is the effect.

#### 2 Deforestation: cutting off the CO_2 _source

Deforestation currently accounts for a significant fraction of the anthropogenic CO_2 _emissions (0.5–2.7 GtC y^-1^; [1]). While mid-latitude regions such as China, India, Western Europe and North America were mostly deforested in earlier centuries, current deforestation takes place mainly in the tropics, notably the Amazon and Southeast Asia. Deforestation at the southern Amazon is typically done at the end of the dry season. Trees are cut, piled up and burned, often with the help of kerosene. While development pressure makes deforestation difficult to stop at present, burying the downed trees instead of burning will reduce the associated CO_2 _emissions at minimum cost. Such a strategy is not in defense of deforestation, but serves to reduce its negative impact.

#### 3 Post-consumer wood: making waste a carbon sink

A large fraction of municipal waste is wood, e.g., old furniture and construction lumber, and backyard dead trees. Most of these are burned or buried in landfills where they may already have relatively long lifetime. If these can be collected and buried in landfills with long-storage time ensured, it will serve as a carbon sink of up to 1 GtC y^-1 ^assuming the current wood use rate [35]. This is of course part of the estimated 10 GtC y^-1 ^world potential. One advantage of burying waste wood is that there will be no additional ecological impact, unlike wood harvest from the forest. Because it already carries significant cost to handle the waste wood, burial for carbon sequestration should be even more economically viable. On the other hand, such wood could also be incinerated to produce energy and their costs and relative merits need to be evaluated, but the wastes do not have to be wasted anymore.

#### 4 Fire suppression: burying the fuel

Fire suppression, such as in the US and Canada over last several decades, has left a large amount of dead vegetation on the forest floor and contributed to an apparent carbon sink in North America. This additional fuel load, combined with recent drought in the America West has led to more frequent and large fires in recent years. The release of this carbon pool through catastrophic fires may become an important source to atmospheric CO_2 _in the future. Collecting dead trees and burying them would reduce fire danger while creating a carbon sink.

## Conclusion

Coal was formed by the burial of ancient plants in anaerobic conditions such as swamp and peatland. The proposed wood burial method is essentially a first step of a fossil fuel formation process, only drastically *accelerated *by active human management. It is ironic that the whole climate change problem is caused by the human *accelerated *release of the fossil fuel carbon pool. Thus it will not be surprising if this method turns out to be the most 'natural' way to undo fossil fuel CO_2 _emission.

The wood burial technique uses natural tree growth to capture CO_2 _from the air at nearly no cost, thus making it significantly more economical than other carbon capture methods. For storage, past focus has been on geological formations and in the ocean. Storing carbon by wood burial under soil will not only cut down atmospheric CO_2_, but also relieve the CO_2 _burden on the ocean where acidification is of major concern [39]. The traditional carbon sequestration techniques tend to be industrial scale, while the present proposal is a distributed approach. This has both advantages and disadvantages that need to be sorted out. It is likely that many of these methods will be practiced to some degree, but the merits of wood burial make it an attractive option: low tech, low cost, distributed, easy to monitor, safe, reversible, thus a no-regret strategy. On the other hand, forest is a precious resource Mother Nature endowed upon us that serves many critical ecosystem functions and human needs. Care needs to be taken in pursuing such a strategy at large scale.

## Competing interests

The author(s) declare that they have no competing interests.

## Authors' contributions

This is a single-authored paper.

## References

[B1] IPCC (2007). Climate Change.

[B2] Canadell JG (2007). Proceedings of the National Academy of Sciences.

[B3] Nakicenovic N (2000). Special Report on Emissions Scenarios.

[B4] Friedlingstein P (2006). Journal of Climate.

[B5] Hansen JE (2005). Climatic Change.

[B6] O'Neill BC, Oppenheimer M (2004). Proceedings of the National Academy of Sciences of the United States of America.

[B7] Schneider SH, Mastrandrea MD (2005). Proceedings of the National Academy of Sciences of the United States of America.

[B8] Stern N (2007). The Economics of Climate Change.

[B9] Pacala S, Socolow R (2004). Science.

[B10] Hoffert MI (2002). Science.

[B11] Schrag DP (2007). Science.

[B12] Brewer PG, Friederich C, Peltzer ET, Orr FM (1999). Science.

[B13] House KZ, Schrag DP, Harvey CF, Lackner KS (2006). Proceedings of the National Academy of Sciences of the United States of America.

[B14] B M et al, IPCC (2005). Special Report: Carbon Dioxide Capture and Storage.

[B15] Zeng N (2003). Advances in Atmospheric Sciences.

[B16] Zeng N (2007). Clim Past.

[B17] Micales JA, Skog KE (1997). International Biodeterioration & Biodegradation.

[B18] Zeng N, Qian HF, Munoz E, Iacono R (2004). Geophysical Research Letters.

[B19] Zeng N, Qian HF, Roedenbeck C, Heimann M (2005). Geophysical Research Letters.

[B20] Friedlingstein P, Joel G, Field CB, Fung IY (1999). Global Change Biology.

[B21] Harmon ME, Brown S, Gower ST, Vinson TS, Kolchugina TP (1993). Carbon Cycling in Boreal Forest and Sub-aritic Ecosystems.

[B22] Matthews E (1997). Journal of Geophysical Research-Atmospheres.

[B23] IPCC (2000). Special Report on Land Use, Land-use Change and Forestry.

[B24] Odum EP (1969). Science.

[B25] (2001). FAO, "Global Forest Resources Assessment 2000".

[B26] Baldocchi D (2001). Bulletin of the American Meteorological Society.

[B27] Gurney KR (2002). Nature.

[B28] Rayner PJ, O'Brien DM (2001). Geophysical Research Letters.

[B29] Visser R (2007).

[B30] Archer D, Kheshgi H, MaierReimer E (1997). Geophysical Research Letters.

[B31] Vitousek PM, Fahey T, Johnson DW, Swift MJ (1988). Biogeochemistry.

[B32] Schlesinger ME (1991). Biogeochemistry: an analysis of global change.

[B33] Galloway JN, Schlesinger WH, Levy H, Michaels A, Schnoor JL (1995). Global Biogeochemical Cycles.

[B34] Hungate BA, Dukes JS, Shaw MR, Luo YQ, Field CB (2003). Science.

[B35] Hurtt GC (2006). Global Change Biology.

[B36] Stephanopoulos G (2007). Science.

[B37] Dyson FJ, Marland G (1979). Workshop on the Global Effects of Carbon Dioxide from Fossil Fuels.

[B38] Sedjo RA (1989). Environment.

[B39] Caldeira K, Wickett ME (2003). Nature.

[B40] Marland G, Boden TA, Andres RJ (2007). Trends: A Compendium of Data on Global Change.

[B41] Sabine CL (2004). Science.

